# Neural bases of hand synergies

**DOI:** 10.3389/fncom.2013.00023

**Published:** 2013-04-08

**Authors:** Marco Santello, Gabriel Baud-Bovy, Henrik Jörntell

**Affiliations:** ^1^Neural Control of Movement Laboratory, School of Biological and Health Systems Engineering, Arizona State UniversityTempe, AZ, USA; ^2^Department of Robotics, Brain and Cognitive Sciences, Istituto Italiano di TecnologiaGenova, Italy; ^3^Faculty of Psychology, Vita-Salute San Raffaele UniversityMilan, Italy; ^4^Neural Basis of Sensorimotor Control, Department of Experimental Medical Science, Lund UniversityLund, Sweden

**Keywords:** degrees of freedom, premotor neurons, manipulation, motor cortex

## Abstract

The human hand has so many degrees of freedom that it may seem impossible to control. A potential solution to this problem is “synergy control” which combines dimensionality reduction with great flexibility. With applicability to a wide range of tasks, this has become a very popular concept. In this review, we describe the evolution of the modern concept using studies of kinematic and force synergies in human hand control, neurophysiology of cortical and spinal neurons, and electromyographic (EMG) activity of hand muscles. We go beyond the often purely descriptive usage of synergy by reviewing the organization of the underlying neuronal circuitry in order to propose mechanistic explanations for various observed synergy phenomena. Finally, we propose a theoretical framework to reconcile important and still debated concepts such as the definitions of “fixed” vs. “flexible” synergies and mechanisms underlying the combination of synergies for hand control.

## Introduction

The structure of the hand, with its intricacy of bones, muscles, tendons, blood vessels, and nerves, is a marvel of evolution yet unsurpassed by any artificial hand. At the functional level, the hand is also a marvel of dexterity and versatility that combines a rich sensory endowment with strength: it holds the scalpel of the neurosurgeon, the pen of the scribe, the brush of the aquarellist as well as the hammer of the blacksmith or the sword of the warrior. One sign of the importance of the hand for humans is its remarkable evolution, with the development of the opposable thumb which underlies all skilled procedures of which the hand is capable (Napier, 1956, 1980) and, at the neuronal level, of the corticospinal tract, which allows the brain to control it in a much more direct way than in other species. The role that the hand plays in almost all our activities and its adaptability to a wide range of behavioral contexts, have led some to see the hand as a simple executant of the commands coming from the higher centers in the brain.

Whereas the motor apparatus of the hand offers a tremendous movement range and adaptability (or, in more technical terms, features a very high number of *degrees of freedom*, DoF), this feature also makes the human hand exceedingly difficult to control—as underscored by the challenges faced by robotics and neuroprosthetics in controlling the latest generation of anthropomorphic hands. The biomechanical and functional characteristics of the hand make it a remarkable model to investigate how the brain controls the many DoFs of the body, one of the fundamental problems of motor control (e.g., Bernstein, 1967; Turvey, 2007; Latash, 2008). As pointed out by many investigators, having a large number of DoFs allows one to perform a task in a wide variety of ways, which presents considerable advantages in terms of flexibility and adaptability. For example, one might grasp an object or distribute the forces holding the object differently depending on specific and possibly contingent events such as holding two cups at once, or grasping them using a broken finger. However, the flexibility afforded by the many DoFs of the hand, which allows it to be used in a wide variety of situations, comes also with the price that the central nervous system (CNS) must control a system that is in general vastly more complex than necessary to execute any particular task. This control problem becomes even more daunting if one considers the entire time course of an action. A simple count of the number of mechanical DoFs like the number of joints or muscles in the hand vastly underestimates the complexity of the problem that the motor system must solve since it amounts to counting the number of parameters necessary to specify the hand state at a precise moment in time. Most actions are dynamic and require a constant and mutual adjustment of all elements of the system. For example, reaching for an object requires transporting the hand while orienting and pre-shaping it, as well as making postural adjustments to keep the body's center of gravity within the base of support defined by the positions of the feet. The number of redundant DoFs increases almost without limits if we consider the temporal evolution of the system rather than a static snapshot of it.

The concepts of synergy and “synergy control” have attracted considerable interest in motor control neuroscience in recent years as a possible solution to this problem. According to Turvey (2007), a synergy is “a collection of relatively independent degrees of freedom that behave as a single functional unit – meaning that the internal degrees of freedom take care of themselves, adjusting to their mutual fluctuations and to the fluctuations of the external force field, and do so in a way that preserves the function integrity of the collection” (p. 659). In other words, a synergy is a functional property of a multi-element system performing an action, whereby many elements of the system are or can be constrained to act as a unit through a few coordination patterns to execute a task. In principle, “synergy control” could combine dimensionality reduction with great flexibility.

The main goal of this review is to illustrate how various analyses of hand motor control point to the fact that the control problem of apparently complex manual tasks is solved by extensive dimensionality reduction and that the number of effective DoFs present in a task might actually be quite small. We will start out by describing the peripheral apparatus and the biomechanical characteristics of the hand that are more relevant to this review. We then review work on kinematic synergies, i.e., tasks involving actual hand movements, and proceed with force synergies, i.e., tasks involving different kinds of grasping and the force equilibrium maintained through the fingertips. In the last section we review evidence for synergy control embedded in the “infrastructure” of the nervous system, i.e., the neuronal circuitry involved in hand movement control. Finally, we conclude with open questions and directions for future research.

## Biomechanical constraints

The hand is first and foremost a complex mechanical structure that includes 27 bones actuated by 18 intrinsic and 18 extrinsic muscles by means of a complex web of tendons. A first measure of the complexity of the hand is to consider the number of joints in the hand. A simple kinematic model of the hand typically consists of four DoFs for each finger, four or five DoFs for the thumb, plus one DoF at the radio-ulnar joint and two DoFs at the wrist, yielding a total of 23 or 24 DoFs. A more detailed kinematic model would include additional parameters to describe arching of the palm that occurs when the thumb tip approaches the tip of the ring or little fingers. A complete biomechanical model of the hand would also include the 36 muscles acting on the thumb and fingers and the complex web of tendons that actuate the hand, yielding total of at least 60 DoFs without even taking into account muscles acting on the wrist and radioulnar joints or additional DoFs associated with contact forces. Therefore, even for seemingly trivial motor tasks such pushing a key with a finger, a complete biomechanical account of the action would require a description of how the combined activation of many muscles contributes to generate desired motions and contact forces.

**Figure 1:**
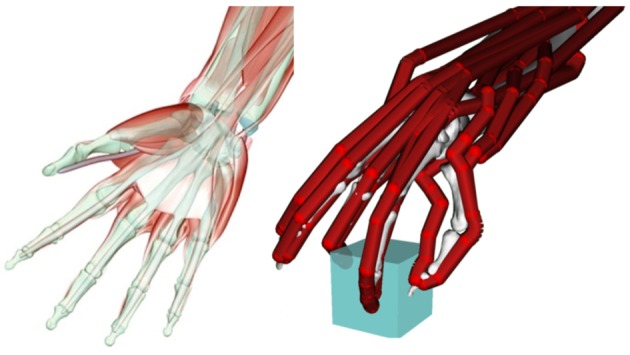
**Left:** muscular and skeletal architecture of the hand. Note the multi-joint tendons of the extrinsic finger flexor muscle (m. flexor digitorum profundus). **Right**: simplified model of a hand grasping an object with three fingertips (tripod grasp). The bones are only vaguely visible in the background, whereas the tendons are indicated in red. Only a subset of the tendons/muscle acting on each finger is included in this model (Holzbaur et al., 2005).

There are several reasons why knowledge of hand biomechanics is important for understanding the neural control of the hand. First, biomechanical constraints might limit the actions of the hand, therefore motor commands must adapt to these constraints to avoid engaging these actions, to find ways around the constraints, or to exploit them. For example, we cannot stick a finger in an S-shaped tube because of the impossibility to fold the two inter-phalangeal joints in different directions. Another constraint is that hand dimensions and digit lengths limit the size of an object that can be grasped with one hand. Secondly, biomechanical constraints can, in principle, reduce the number of independent DoFs. For example, extrinsic finger flexor and extensor muscles have tendons that span several joints of each finger (see Figure 1). Therefore, assuming a perfectly focal activation of a single muscle compartment (e.g., the index finger compartment of *m. flexor digitorum profundus*), contraction of that muscle would cause flexion at several joints. This partly accounts for the difficulty encountered when trying to move only the distal phalange of a finger without also moving the more proximal joints. It should also be noted that the biomechanical architecture constraining coupled actions at multiple joints *within* a digit also characterizes coupled actions *across* digits. Specifically, interconnections between tendons of hand muscles and long tendons spanning all finger joints limit the extent to which focal activation of hand muscles innervating a finger can isolate torque generation at one joint or at a given finger from torque generation in adjacent fingers [see Schieber and Santello (2004), for review of peripheral constraints on hand function]. Third, what might appear like simple movement, e.g., flexion of one finger around one joint, might require the coordinated action of several hand muscles (Reilly and Schieber, 2003; Schieber and Santello, 2004). Fourth, and most importantly, biomechanics is crucial to understanding the mechanical functions of a muscle and how motor commands adapt to task conditions. For example, finger posture has been shown to significantly affect the mapping between muscle activation and isometric joint torque production (Kamper et al., 2006).

This brief description of the hand anatomy should make clear that the brain does not directly control the movement of the individual muscles or joints of the hand. Therefore, the biomechanical structure of the hand defines a complex mapping between the motor commands and the observable motions or contact forces at the digits.

## Kinematic synergies

In the past 20 years, hand kinematics has been studied in a variety of tasks. Despite significant differences in the requirements of these tasks, all of these studies share a main observation: simultaneous motion of multiple digits occurs in a consistent fashion, even when the task may require a fairly high degree of movement individuation such as grasping a small object or typing.

### Covariation of finger movements

The word “synergy” has been used in several contexts when describing thumb and finger movements, often referring to qualitative observations of movement patterns that are characterized by simultaneous motion of the fingers in the early stages of development (palmar grasp reflex) and for the purpose of classifying hand movements (Elliott and Connolly, 1984) or grasp postures along a functional gradient (Napier, 1956, 1980; Cutkosky, 1989). The first attempts at quantifying the kinematics of hand synergies focused on the spatial and temporal coordination of digit movements in serial tasks requiring isolated motion of one digit, i.e., typing (Fish and Soechting, 1992; Flanders and Soechting, 1992; Angelaki and Soechting, 1993; Gordon et al., 1994; Gordon and Soechting, 1995; Soechting and Flanders, 1997), and later extended to movement sequences involving motion of one or more digits [in piano playing and finger spelling (Engel et al., 1997; Jerde et al., 2003a,b)]. These studies found that when subjects typed a single letter with a finger (“focal movement”), motion that was not necessary to complete the task (“corollary motion”) also occurred at other fingers in a subject-dependent but stereotypical fashion. These studies also reported that the degree of correlation of movement across pairs of digits—stronger for adjacent than non-adjacent digits and higher when neither of a pair of fingers is used to strike the key—was not obligatory, hence not uniquely due to biomechanical constraints (Fish and Soechting, 1992; Angelaki and Soechting, 1993; Soechting and Flanders, 1997). These early observations led to the suggestion that “… synergistic finger motions would simplify the problem of controlling the large number of DoFs inherent in motion of all of the fingers of the hand …” (Fish and Soechting, 1992). A subsequent typing study further revealed that only a few principal components (PCs) could characterize motion of a subset (17) of all kinematic DoFs of the hand, thus implying “… a reduction in the number of dofs independently controlled by the nervous system” (Soechting and Flanders, 1997) stemming from musculoskeletal and neural constraints. The consistency with which these constraints operate significantly facilitates hand shape recognition in finger spelling due to “leakage” of information across finger joint angles (Jerde et al., 2003a). Similarly to what was found for typing movements, however, covariations in finger joint excursions exhibit some degree of task-dependency as indicated by the sensitivity of hand shape to the preceding or following letter (Jerde et al., 2003b). A recent application of PC analysis has been introduced to study sensorimotor transformations by quantifying humans' ability to perceive and reproduce hand postures with the contralateral hand as a function of grasping force and forearm orientation relative to gravity (Pesyna et al., 2011).

### Dimensionality reduction in the analysis of finger movements during reach-to-grasp

Research on synergy control has been devoted to developing analytical techniques to reveal if and how a reduction in the dimensionality of the hand control space is attained. In this section we discuss the reduction in dimensionality of hand kinematics defined as joint angles observed during grasping and reach-to-grasp movements.

The concept of synergies, often quantified and defined through dimensionality reduction techniques [principal components analysis, PCA; singular value decomposition, SVD; non-negative matrix factorization, for review see Tresch et al. (2006)], has also been invoked when describing systematic covariations of joint angular excursions of hand postures used for grasping. The first description of hand postural synergies for grasping movements (Santello et al., 1998) was based on grasping a large number of imagined objects with different sizes and shapes. This design was motivated by the fact that hand shape at contact with an object results from central planning as well as the mechanical interaction of the hand with the object. By asking subjects to shape the hand to imagined objects, one could examine the central planning of hand posture as a function of object shape. Subsequent work on grasping real objects (Santello et al., 2002) confirmed the main observations made on hand posture used for grasping imagined objects by revealing, as expected, a larger number of PCs when physical contact was allowed.

Similar to the results of PCA of typing movements (Soechting and Flanders, 1997), the main finding of the study by Santello et al. (1998) was that a few linear combinations of the 15 DoFs that were measured could account for most of the variance in the original set of hand postures. The lower order components (PC1-3) described more “basic” patterns of finger motion, e.g., hand opening/closing caused by motion at all metacarpal-phalangeal or proximal-interphalangeal joints. Interestingly, however, hand shapes reconstructed by adding higher order PCs (up to the fifth or sixth) provided additional information about the object. These observations led to the suggestion that the control of hand posture might be implemented by a combination of postural synergies ranging from those responsible for the general shape of the hand (lower PCs) to those responsible for subtler kinematic adjustments (Santello et al., 1998). A similar framework was also proposed when interpreting the results of an earlier study on matching haptically or visually perceived object size (Santello and Soechting, 1997). As noted by the authors, PCs do not need have any physical significance, and therefore they cannot be used to infer the relative contribution of peripheral constraints vs. central commands in generating coupled motion of the digits. However, transcranial magnetic stimulation (TMS) of primary motor cortex can elicit synergistic finger movement patterns similar to those found when the same subjects grasped imagined objects (Gentner and Classen, 2006), suggesting a modular organization of cortical representations of hand muscles. Nevertheless—and as noted above for finger movement patterns in typing—covariation patterns of finger motion are not obligatory. This implies that hand synergies revealed by the above studies are not a mere byproduct of anatomical factors such as multi-tendoned and multi-joint muscles, and therefore that the CNS retains the ability of partially overriding, in a task-dependent fashion, anatomical constraints. Specifically, finer modulation of neuromuscular activity might be required to overcome peripheral coupling when the task requires independent finger actions (Santello et al., 1998) or to exploit it when multi-digit actions require all digits to act as a unit (Schieber and Santello, 2004). These considerations also explain why manipulations characterized by different requirements in the spatio-temporal coordination of the digits can elicit different number and patterns of PCs (Todorov and Ghahramani, 2004). Similarly, task-dependencies in finger joint angle covariations have also been reported when comparing whole-hand grasping of one object vs. individuated finger movements (Braido and Zhang, 2004). However, the relatively large number of PCs associated with haptic exploratory procedures can be used to reconstruct grasp postures, indicating some commonalities of digit movement coordination patterns across these tasks (Thakur et al., 2008).

Hand kinematics and coordination patterns of multi-digit motion, although not always quantified or defined as synergies in a lower dimensional space, have also been examined in terms of the temporal evolution of hand shape during reach-to-grasp as a function of object geometry (Santello and Soechting, 1998), sudden changes of either object shape (Ansuini et al., 2008), gravitational conditions (Micera et al., 2002), task in healthy subjects (Mason et al., 2001; Ansuini et al., 2006, 2008) and neurologically impaired individuals (Schettino et al., 2004, 2006, 2009; Ansuini et al., 2010), and sensory feedback in humans (Santello et al., 2002; Schettino et al., 2003; Winges et al., 2003) and non-human primates (Mason et al., 2004; Theverapperuma et al., 2006). These studies have characterized tendencies in task and/or sensory modality-specific kinematic coordination patterns. For example, it has been shown that online visual feedback of the hand and/or object are not necessary for whole-hand shaping to object geometry (Santello et al., 2002; Mason et al., 2004) but it might nevertheless be used to modulate finger motion in the late portion of the reach (Schettino et al., 2003). Another important observation is that the temporal evolution of hand posture to object shape in monkeys occurs through a stereotypical temporal coordination of finger motion superimposed on a movement amplitude scaling (Theverapperuma et al., 2006). More recently, dimensionality reduction in hand control has been described during bimanual grasping (Vinjamuri et al., 2008) and while learning cursor control through finger movements (Vinjamuri et al., 2009). These authors have also used PCA and SVD analyses to characterize digit joint velocities of grasping movements performed at natural (Vinjamuri et al., 2007) and fast speed (Vinjamuri et al., 2010a,b).

In summary, all these studies of hand kinematics point to the same, main conclusion: the dimensionality of the kinematic space of a large repertoire of hand behaviors is significantly smaller than that defined by the number of the hand's mechanical DoFs.

## Force synergies

Grasping can be defined as holding an object stationary within the hand^1^. Precision grasps predominantly involve the fingertips while power grasps usually involve contact with more extended parts of the hand such as the palm. Grasp planning requires that one selects a grasp (e.g., whether to use a precision or power grasp) as well as the position of the contact points on the object for a particular grasp. In precision grasps, the position of the fingertips on the object plays an important role in determining the net force/torque that can be produced. It should be noted that some choices might be incompatible with the task constraints. For example, it might be impossible to use a pinch grasp to lift a very slippery object. Alternatively, multiple grasps may be compatible with task demands. Finally, some choices might lead to desirable properties of the grasp. For example, force closure ensures that it is possible to produce an arbitrary net force and/or torque while form closure ensures a stable grasp in the absence of friction forces (Bicchi et al., 2011).

Once a contact is established, a precise control of the contact forces with the object and/or net force is necessary in order to be able to perform any skilled manipulation. For example, when holding an object immobile in the air, the net force must balance exactly the gravitational force. Similarly, the use of most tools requires a precise control of interaction forces. While a complete mechanical analysis of the finger forces during this phase is outside the scope of this review (see Inset 1), task and frictional constraints do not, in general, fully specify the contact forces. In other words, from a control point of view, there is usually an infinite number of ways of setting the contact forces that are compatible with all constraints (Murray et al., 1994; Li et al., 1998; Zatsiorsky et al., 2003a).

Inset 1 **Task constraints and degrees of freedom in the control of contact forces.**

Force synergies reflect coordination patterns at the level of the contact forces. This inset gives an overview of the contact forces involved in precision grasps in order to clarify the parameters that need to be controlled and the task constraints that act at this level. Contacts in precision grasps involve small areas of the hand such as the fingertips and can be modeled as a force applied at a point on the object together with a moment along the normal of the contact surface [the so-called *soft finger model*, (Murray et al., 1994)]. According to this model, four parameters are needed to describe each contact. More complex models accounting for the geometry and/or compliance of the finger pads have been developed in robotics but are outside the scope of this article as they are rarely used to analyze human grasps.

**Figure 2:**
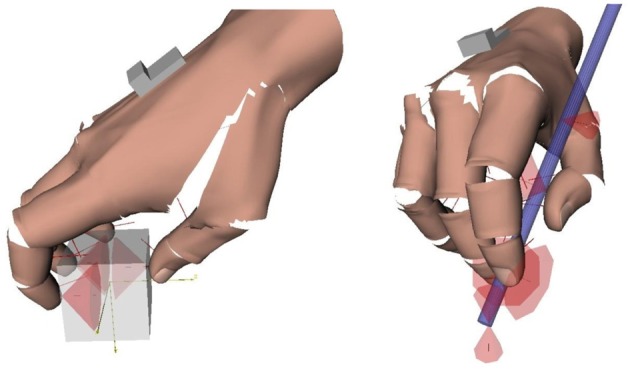
**Contacts in precision grasps. Left**: example of multi-digit (tripod) grasp invovling only contacts with the fingertips. Contacts are represented by their friction cones. **Right**: hand-object contacts in the pen grasp. The friction cones at the extremity of the pen represent the contact with the table. The computation of the contacts and graphical rendering was done with Grasp-It, an open-source software dedicated to the analysis of grasps (Miller and Allen, 2004).

When grasping and manipulating an object, *task constraints* define the net force and moment. For example, when holding an object immobile in the air, the net force must oppose the gravitational force and the net moment must be 0. To satisfy these constraints, the contact forces must satisfy *equilibrium equations* that relate the finger forces to the net force and moment. In addition, each contact force must also satisfy a *frictional constraint* which specifies that the normal force (i.e., the force component perpendicular to the contact surface) must be larger than the tangential force (i.e., the force component parallel to the contact surface) divided by the coefficient of friction of the contact surface to avoid a slip of the finger. Geometrically, this constraint states that the contact force must be in the so-called friction cone, the aperture of which depends on coefficient of friction of the surface (see Figure 2). Often, this constraint implies that one must squeeze the object with more force to increase the normal forces when the load (i.e., the tangential force) increases.

In the analysis of contact forces, the complexity of the control task is often defined in terms of the number of redundant DoFs, i.e., the number of DoFs that are not constrained by the task. In general, the number of redundant DoFs increases with the number of digits involved in the grasp. For example, in precision grasps where the structure of the hand allows control of the direction and magnitude of the force at each fingertip independently, there are at least 3 redundant DoFs in a tripod grasp (left panel of Figure 2) and 9 redundant DoFs in a five-digit grasp. This high degree of redundancy raises the question of how the brain selects one solution among all possible ones (Bernstein, 1967).

Counting the number of degrees of freedom is not without difficulties however (see also Introduction). In the analysis of force synergies, the number of DoFs of the grasp is often defined as the total number of parameters needed to specify the contact forces. However, this definition assumes that all parameters can be controlled independently, which is not always the case. For example, when a finger makes two contacts with an object, it is impossible to control the direction and magnitude of the two contact forces independently. Similarly, when holding a pen, the structure of the hand does not afford control of the contact force between the pen and the palm independent from the contact force between the fingertips and the pen (see right panel of Figure 2). In these cases, a more complex analysis of the structure of the hand is necessary to identify the effective number of DoFs.

The extent to which the CNS can control finger forces independently has been studied in multi-finger pressing tasks where one must produce force by pressing on a surface with one or more fingers. In these studies, a sensor measured the force produced by each finger while subjects were instructed to produce certain levels of force. A common observation is that all fingers produced forces even when the participant was instructed to apply a force with only one finger (Li et al., 1998; Reilly and Hammond, 2000, 2004; Zatsiorsky et al., 2000). This phenomenon, called *enslaving*, is in agreement with the observation that it is difficult to move one finger without moving the others [Kilbreath and Gandevia, 1994; Hager-Ross and Schieber, 2000; Lang and Schieber, 2003; Kim et al., 2008; see above *Kinematic Synergies* section].

In tasks involving grasping, lifting, and holding an object against gravity with five digits, the variations of the contact forces while holding an object have also been analyzed to understand the spatial and temporal coordination of multi-digit forces. For example, Santello and Soechting (2000) described consistent in-phase relations between pairs of digit normal forces. These coordination patterns, also found regardless of hand dominance or object property predictability (Rearick and Santello, 2002), could theoretically be dismissed as a by-product of biomechanical factors constraining the temporal relations among digit forces. However, a subsequent study revealed these patterns to be task-dependent as they disappear when the same forces are applied to the object resting on a table as opposed to being held against gravity (Rearick et al., 2003). These findings suggest an active, neural component compensating for temporal fluctuations in multi-digit forces to satisfy the above-described constraints of mechanical equilibrium.

A striking observation in force production and grasping tasks is the high amount of inter-trial variability present in these tasks. For example, in multi-finger force production tasks where the total force is constrained, the normal forces produced by each finger vary much more than the total force (Latash et al., 2001). In these tasks, fingers clearly act synergistically to produce a stable performance.

### Hierarchical control of contact forces

To explain force synergies observed in grasping, a general idea is that contact forces are controlled hierarchically. In other words, higher-level constraints might impose coordination patterns between the contact forces.

Latash and collaborators have proposed that the variables controlled by the CNS are not the individual finger forces but the *force modes*, i.e., force patterns distributed across fingers reflecting the enslaving phenomenon in force or grasping tasks (Danion et al., 2003). Their analysis focused on the normal component of the contact forces and they assumed the existence of five force modes, one per finger. The instruction of pressing against a surface with only one finger would presumably activate one mode that primarily controls the instructed finger. Different force patterns required in other tasks would be obtained by activating simultaneously two or more force modes. Follow-up studies extended this idea to various grasping tasks (Zatsiorsky et al., 2003b). Conceptually, the force modes correspond to a re-parameterization of the finger force parameters but it is not clear whether they actually reduce the number of parameters that the CNS needs to control. Moreover, a comparison between the results obtained from the same individuals in pressing and grasping tasks showed that force modes could be both task-specific (Olafsdottir et al., 2005) and subject-dependent (Gao et al., 2003). This flexibility, however, raises the question of whether controlling an alternative set of variables (the force modes) to control contact forces is actually simpler than controlling contact forces directly. Therefore, one might as well look for multi-digit synergies not at the level of force modes, but at the level of the components of the contact forces themselves [(Latash, 2008), p. 207].

The analysis of the variability in motor tasks by the same group of researchers led them to formulate the *uncontrolled manifold (UCM) hypothesis*, which they have used to discover and quantify synergies (Scholz and Schoner, 1999; Scholz et al., 2003; Kang et al., 2004). The hierarchical nature of the UCM hypothesis is reflected in the distinction between *elemental* and *performance* variables. Elemental variables are loosely defined as related to the parts of the system (e.g., components of the contact forces) while performance variables are related to the task (e.g., production of a given total force across multiple digits). This classification is central to establishing another distinction between the so-called bad variance (VB), i.e., variability of the elemental variables that would lead to a loss of precision in the task, and good variance (VG), i.e., variability of elemental variables that would compensate each other and thus not affect the performance. A large ratio VG/VB would be the sign of a strong synergy while a smaller ratio would correspond to a weak synergy.

Another proposal to explain the observed coupling between finger forces is the *virtual finger (VF) hypothesis*. This hypothesis proposes that the brain controls the position and force of one or more VFs at a higher level of the control hierarchy (Iberall et al., 1986; Baud-Bovy and Soechting, 2001; Shim et al., 2003; Zatsiorsky et al., 2003b; Smith and Soechting, 2005). At a lower level, the forces produced by the physical fingers would be coordinated to match the constraints induced by the VF(s). Unlike the force mode hypothesis, the VF hypothesis does not determine a fixed pattern of covariation between the contact forces because the constraints induced by the VF(s) still leave redundant DoFs in the grasp. For example, the VF hypothesis in the tripod grasp can account for the coupling between contact forces of the index and middle fingers, but does not fully specify their directions, which might be optimized to increase the stability of the grasp as a function of object shape (Baud-Bovy and Soechting, 2001).

One issue with hierarchical control schemes is that higher-level constraints do not in general specify all parameters at the lower level, which raises the question of how remaining parameters are selected (see inset 1). One partial answer is that these parameters might be selected so as to maximize the stability and efficiency of the grasp. More generally, the core idea of optimal control is that behavior reflects the optimum of some cost function (Todorov and Jordan, 2002; Todorov, 2004). In the context of grasping, this general framework has been used to explain both how a set of contact positions (Friedman and Flash, 2007; Lukos et al., 2007, 2008; Ciocarlie et al., 2009; Fu et al., 2010, 2011; Craje et al., 2011; Gilster et al., 2012) and contact forces (Hershkovitz et al., 1997; Chalfoun et al., 2004; Pataky et al., 2004; Baud-Bovy et al., 2005; Niu et al., 2009; Terekhov et al., 2010) are selected. However, a problem with this framework is that it does not explain how the optimal solution might be actually computed in a biologically plausible manner.

Another possibility might be that the CNS does not control all parameters of the grasp (e.g., contact forces, finger positions, net force, and torque). Actually, this might be excessively difficult to do because it would require the CNS to have an inverse model of the complex biomechanical structure of the hand in order to activate the muscles to obtain a very specific level of force at each contact point to satisfy the equilibrium equations. Instead, the CNS might rely on the compliance of the fingers to balance the contact and external forces. In this case, the coordination patterns observed between the contact forces could reflect both the passive properties of the hand-holding-an-object system and the active contribution of the CNS (Ostry and Feldman, 2003; Winges et al., 2007; Gabiccini et al., 2011). Recent work has been conducted to re-interpret multi-digit synergies in the framework of the equilibrium point hypothesis (Pilon et al., 2007; Latash et al., 2010).

## Muscle synergies

The hypothesis that movements are generated by combining a small group of muscles has been extensively studied since the early observations of Sherrington (1910) on the wide range of reflex-mediated movement patterns in the cat. These combinations are generally referred to as “muscle synergies” [for recent reviews, see Ting and McKay (2007), Tresch and Jarc (2009)]. Whereas the number of muscles acting around one or more joints is finite, the number of muscle synergies can theoretically be very large when considering modulation of the timing at which muscles can be activated relative to each other and/or the number of motor units that can be recruited in a given muscle. The timing of muscle and motor unit activation is best measured by direct recordings of their activity through electromyographic (EMG) recordings.

### EMG and the study of muscle synergies in the hand

EMG has been extensively used as a tool to study the spatial and temporal coordination of multiple muscles. The characteristics of the EMG signals recorded from active muscles, e.g., its magnitude, frequency content, and/or timing, all reflect the net output of the interactions of neural inputs to the spinal alpha-motorneurons (alpha-MNs). Therefore, EMG signals recorded from muscle fibers innervated by the motor nuclei of hand muscles have been studied to determine the organization and plasticity of the neural drive responsible for coordinating multiple hand muscles during individual finger movements or multi-digit movements, e.g., grasping and manipulation.

The divergence of inputs to motor units of hand muscles has been quantified by measuring temporal and/or frequency correlations in EMG signals (motor-unit synchrony and coherence, respectively) at the single unit level or as multi-unit interference EMG [for review see Santello (in press)], as well as by measuring correlations in the magnitude of EMG signals across muscles acting on one (e.g., Valero-Cuevas et al., 1998; Valero-Cuevas, 2000), or two digits during force production (Maier and Hepp-Reymond, 1995), and three digits during object hold (Danna-Dos Santos et al., 2010; Poston et al., 2010). The existence of these correlations is interpreted as denoting common inputs to hand motor nuclei, and therefore could be considered as a manifestation of “building blocks” of muscle synergies. Correlations in EMG amplitude of hand muscles, quantified through PC analysis, have also been described for whole-hand grasping (Weiss and Flanders, 2004) as well as for tasks that do not involve contact forces, e.g., finger spelling (Weiss and Flanders, 2004; Klein Breteler et al., 2007). In non-human primate models, covariation in EMG amplitude of hand muscles has been described when reaching to grasp objects with different shapes (Brochier et al., 2004). A recent study provided further evidence for muscle synergies in a non-human primate model by revealing the existence of a small number of EMG synergies that could capture the variance of EMG activity patterns elicited by grasping objects of variable shapes and sizes (Overduin et al., 2008). A follow-up study further revealed that similar postures could be elicited by electrical microstimulation to motor cortical areas (Overduin et al., 2012).

Human studies using analyses of EMGs from all muscles inserting on the index finger during force production in different directions revealed that the variance-per-dimension (one for each of the 7 muscles) was smaller in the task-relevant subspace than in the task-irrelevant subspace (Valero-Cuevas et al., 2009). This finding supports the “principle of minimum intervention” or optimal sensorimotor control (Todorov and Jordan, 2002; Todorov, 2004), hence compatible with the earlier framework of synergies proposed by Bernstein (1967) (see also above-discussed uncontrolled manifold hypothesis). However, the non-negligible variance in all seven dimensions was also interpreted as evidence against the framework of muscle activation as resulting from the combination of a small set of synergies. A recent study further revealed that EMG amplitude of intrinsic muscles is modulated in tandem with that of extrinsic muscles when holding an object at different wrist angles, even though wrist posture changes the length and moment arm of extrinsic muscles only (Johnston et al., 2010a). The mechanisms underlying these covariations and the cost function(s) being minimized through these coordination patterns remain to be understood. Nevertheless, these findings indicate that the CNS consistently exploits a small set of solutions (motor commands) among many equally valid ones. The question of the extent to which the above-described covariation patterns of EMG amplitudes can be flexibly modulated to task conditions or, conversely, reflect relatively rigid neural constraints, is addressed in the Discussion.

### Interpretation of synergies in EMG recordings

When examining the activity of concurrently active motor units, the nature of these common inputs can be further distinguished depending on the lags between action potentials, near-synchronous discharges being indicative of shared inputs from branched axons of single last-order neurons (short-term synchrony) and longer lags denoting synchrony of separate presynaptic inputs to the alpha-MNs (Kirkwood, 1979). Inferences about the mechanisms responsible for coherence between spike trains of motor units (EMG-EMG coherence) can be made based on the range of frequencies across which significant correlations occurs [i.e., periodic or non-periodic inputs; (Halliday and Rosenberg, 2000; Taylor and Enoka, 2004)], coherence strength (e.g., Johnston et al., 2005, 2010b; Winges et al., 2006), as well as by quantifying the relation between motor-unit synchrony and coherence (e.g., Semmler et al., 2003; Johnston et al., 2005) [for review, see Grosse et al. (2002)]. Motor-unit synchrony has been reported to occur both within and across muscle compartments of finger flexor muscles (i.e., Hockensmith et al., 2005; Winges et al., 2008) and extensor muscles (Keen and Fuglevand, 2004). Furthermore, in static grasp tasks involving multi-digit force coordination, e.g., object hold, it has been found that common neural input is heterogeneously distributed across hand muscles. Specifically, extrinsic hand muscles (e.g., long flexors of the fingers) tend to receive stronger common input than intrinsic hand muscles (Winges and Santello, 2004; Johnston et al., 2005; Danna-Dos Santos et al., 2010; Poston et al., 2010).

**Figure 3:**
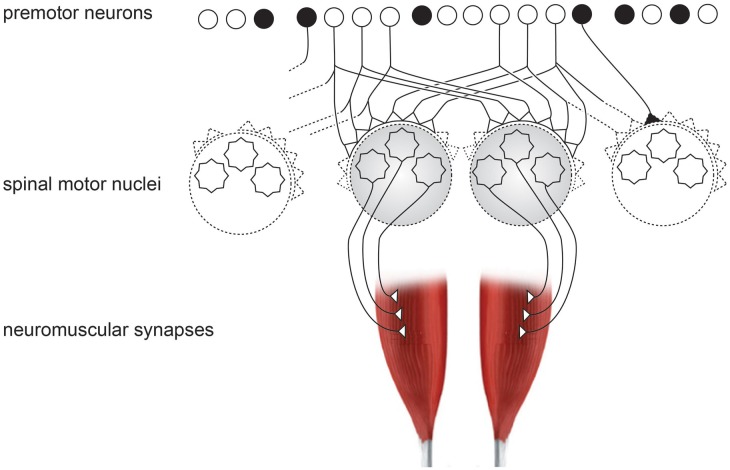
**Schematic organization of neural inputs from premotor neurons to spinal motor nuclei of two hand muscles.** Black neuron represents the inhibitory interneurons, which exist in high numbers.

It should be noted that alpha-MNs are very large neurons, receiving thousands of synaptic inputs on their dendritic tree. Individual synaptic inputs generally have an amplitude of 0.1 mV or less (Asanuma et al., 1979), meaning that the effect of inputs from a single afferent neuron is normally negligible. It follows that synergistic muscle activation requires that the corresponding alpha-MNs share direct inputs from corticospinal tract neurons as well as indirect inputs through a relatively large number of spinal premotor neurons and that these inputs are activated in a concerted fashion (Figure 3). Due to the huge number of synaptic inputs to alpha-MNs and the stochastic properties of the spike firing in alpha-MNs (Moritz et al., 2005), it is difficult to establish the exact mechanisms underlying the moderate strength of synchrony across motor units of different hand muscles, or the difference in the extent of across-motor unit synchrony between long finger flexors and intrinsic muscles such as first dorsal interosseus and first palmar interosseus (Winges et al., 2008). Specifically, EMG recordings can only capture the *net* effect of excitatory and inhibitory inputs, while precluding a clear distinction between these two types of inputs. However, interestingly, synchronized discharge in alpha-MNs, at frequencies which are not present in the excitatory input signal to these neurons, may be generated for example by Renshaw inhibitory interneurons (Williams and Baker, 2009) or possibly other types of spinal inhibitory interneurons driven by Ia afferents (Jankowska, 1992).

## Neural synergies

In the previous sections, we have presented numerous behavioral and physiological observations indicating that the CNS operates in terms of synergy control with respect to the coordination of multi-digit movements and forces. Do the properties of the underlying neuronal circuitry support this notion? Whereas it is indisputable that activation of a given hand muscle generates torque across more than one joint through multi-joint tendons and passive linkages, it is less well-appreciated that in terms of neural control, activation of hand muscle synergies essentially seems to be an inevitable consequence of the known neural connectivity patterns. As we will present below, given that the “infrastructure” of the neuronal connectivity defines the lower bound of what can be achieved in terms of individuated muscle control, it is very difficult to see how the brain could possibly control the hand on a muscle-per-muscle basis.

### Arrangement of hand motor nuclei in the spinal cord vs. neuronal connectivity

An important indicator of the nature of the neural control of the hand is the anatomical distribution of the motor nuclei innervating hand muscles. In a system in which there is a point-to-point innervation of single muscles, one would expect that the motor nuclei of individual muscles would lie separated in the nervous tissue, similar to the oculomotor nuclei in the brain stem. Conversely, in a system in which multiple muscles as a rule are controlled as synergies, the motor nuclei would be expected to lie closely spaced, perhaps even partly overlapping. A relatively complete study of the distribution of hand muscle motor nuclei exists only for the cat (Fritz et al., 1986a,b) but the general findings seem applicable also to the monkey (Jenny and Inukai, 1983). These studies clearly show that the motor nuclei of individual hand muscles are extremely narrow in the transverse plane (i.e., in the cross-section plane of the spinal cord where the motor nuclei are only 2–4 alpha-MNs across) and that the alpha-MNs of different hand muscles are densely packed with the dendrites of the neurons partly extending into the adjacent nuclei containing the alpha-MNs of other muscles. In the longitudinal plane, these motor nuclei form extremely elongated structures, with a substantial overlap in the rostrocaudal extent of the different motor nuclei (Fritz et al., 1986a,b). Considering the wide distribution of the termination territories of single premotor axons (corticospinal and spinal interneurons) in both cat and monkey (Shinoda et al., 1979, 1981; Jankowska, 1992) in the transverse plane, the anatomical structure of the neuronal network seems to be a construct that would promote synergy control.

### Divergent connectivity in motor pathways supports synergy control

In addition to having a divergent innervation to target numerous alpha-MN pools (Shinoda et al., 1981), the majority of the corticospinal terminations in primates are made inside the population of spinal premotor interneurons in laminae VI–VIII (Bortoff and Strick, 1993), where they may be expected to primarily target spinal interneurons. Furthermore, the spinal interneurons of both cat and monkey branch in their innervation of the alpha-MN pools (Jankowska, 1992; Perlmutter et al., 1998; Takei and Seki, 2008, 2010) and hence may add an additional divergence factor on top of the divergent corticospinal terminations (Cheney et al., 1985). Although direct corticomotoneuronal connections have been the focus in the literature of hand muscle control, it is clear that indirect effects, presumably by way of the spinal interneurons, dominate the muscle activation from a single motor cortex cell (Schieber and Rivlis, 2007). In primates, although monosynaptic corticomotoneuronal effects are evident in alpha-MN, indirect effects mediated via the spinal interneurons can be more powerful (Isa et al., 2006, 2007; Alstermark et al., 2007, 2011). Furthermore, the neurons contacting individual hand muscles have a wide distribution across the motor cortex, which heavily overlaps the distributions of neurons controlling other hand muscles (Rathelot and Strick, 2006).

### Spinal premotor machinery—embedded motor functions and synergy control

Much of the literature on neural control of hand movements typically takes its origin in the motor cortex. However, the field of motor cortex research, which has focused on finding correlations between motor cortex neuron discharge and physical parameters of the movement controlled, has failed to reach a consensus of what the functions of the individual neurons are. A possible caveat, which may explain why no consistent pictures have emerged, is that perhaps the neural code in motor cortex does not code for any physical parameter directly, but may rather be a compound code to be deciphered by the downstream neurons in the target region of the spinal cord.

Even for the simplest hand movements, neurons distributed over a large part of the motor cortex are activated (see for example, Schieber and Hibbard, 1993; Georgopoulos et al., 2007) and a single neuron is typically found to be engaged in a wide variety of movement types (Schieber and Hibbard, 1993; Poliakov and Schieber, 1998, 1999; Schieber and Rivlis, 2007). One interpretation of these findings is that in order to generate a movement, the motor cortex needs to control a large proportion of the spinal interneuron pool, which determines the excitability level of most or all of the alpha-MNs innervating the muscles within the arm and hand. Since spinal premotor neurons are strongly driven by peripheral feedback from Ia muscle afferents, Ib tendon organ afferents, and skin sensor afferents (Jankowska, 1992), they cannot be left uncontrolled during any movement or any phase of the movement since the outcome in terms of alpha-MN activation may be unpredictable. By allowing high excitability in *some* interneurons, these types of sensory feedback can instead be utilized by the CNS to become an integral part of the motor command, since the feedback during a given type of movement will become quite predictable as a specific movement pattern is established.

Interestingly, spinal premotor neurons, in addition to innervating alpha-MN nuclei, as a rule have a recurrent axon collateral that either go all the way up to the lateral reticular nucleus for transmission to the cerebellum as mossy fibers (Clendenin et al., 1974a,b; Alstermark et al., 1981; Ekerot, 1990) [including spinal neurons below the C3–C4 segments (Ekerot, 1990)], or to a local projection neuron that issues ascending axons that directly issue mossy fibers to the cerebellum (Oscarsson, 1973; Mrowczynski et al., 2001). This gives the cerebellum direct information about the involvement of the spinal premotor pool. Since it is likely that these premotor neurons are involved in the selection of local motor programs (Grillner, 2003) and thereby synergies, the cerebellum will also be informed about the synergies employed. Whether the implication is that the cerebellum has a role in the learning of muscle synergies is an interesting issue for future studies.

Detailed functional analysis indicates that the spinal cord is to be regarded as a full motor system in its own right (Raphael et al., 2010; Arber, 2012) and its circuitry can play a pivotal role in hand dexterity (Kinoshita et al., 2012). By using the spinal interneuron system, descending motor commands can act on a complex neuronal machinery that comes with a number of features that can facilitate motor control of the complex structure of the extremities. In line with this view, spinal interneurons are activated in advance of onset of movement and alpha-MN activation (Maier et al., 1998; Perlmutter et al., 1998; Prut and Fetz, 1999; Fetz et al., 2002), which can be interpreted as a step to set up the dynamics of this circuitry in advance of the start of the movement. By using the dynamics of the subcortical circuitry the motor cortex could be relieved of solving control issues at a high level of detail. An essential part of this semi-automatic control system is the peripheral feedback from the various sensors of the muscles, joints, and skin. However, although the spinal cord neuronal circuitry has been extensively studied (Jankowska, 1992; Kitazawa et al., 1993; Hultborn, 2001), the complexity of this network has so far prevented us from obtaining a detailed picture of its complete structure in terms of connectivity and function. This remains an important outstanding question for future research on brain synergy control.

## Discussion

The many DoFs of the hand combined with its dexterity and versatility would suggest that the hand must be exceedingly complex to control. On the other hand, as seen in the previous sections, the analysis of the movement of the hand and contact forces has revealed that the DoFs of the hand are typically correlated at every level of description. It should be noted that it is often possible to reduce the observed behavior to the combination of a few basic patterns (see Kinematic and Force Synergies sections). Similarly, the analysis of muscular activities has shown evidence of a common drive between motor units of different muscles. Altogether, these observations indicate that synergy control is a pervasive element of hand function.

However, it should be noted that, in the literature, synergies have been defined in ways that reflect the level at which analysis is performed, even though all definitions point to a reduction in the dimensionality of the control space. Specifically, at the *kinematic level* synergies have been defined as covariation patterns among digit joints during reach-to-grasp and manipulation tasks. At the *kinetic level*, synergies denote coordination patterns among digit forces that are thought to minimize given cost functions. At the *neural level*, synergies consist of common divergent inputs to multiple neurons.

In this section, we propose a control scheme where the spinal circuitry would play a central role in explaining the observed coordination patterns. This proposed scheme could also explain a long-standing question in biology about whether synergies are flexible or fixed. Even during simple hand movements, cortical activation involves large parts of hand motor areas in the primary motor cortex, which via divergent direct and indirect connections would result in excitatory drive to a large proportion, most likely all, of the alpha-MNs innervating all the muscles of the lower arm. However, for any given alpha-MN, the output activity depends on the balance of excitatory and inhibitory inputs from the premotor neurons. As a consequence, inhibitory spinal premotor neurons, the only element providing synaptic inhibition to alpha-MNs, may play a key role in the actual selection of the muscles that will *not* be activated.

**Figure 4:**
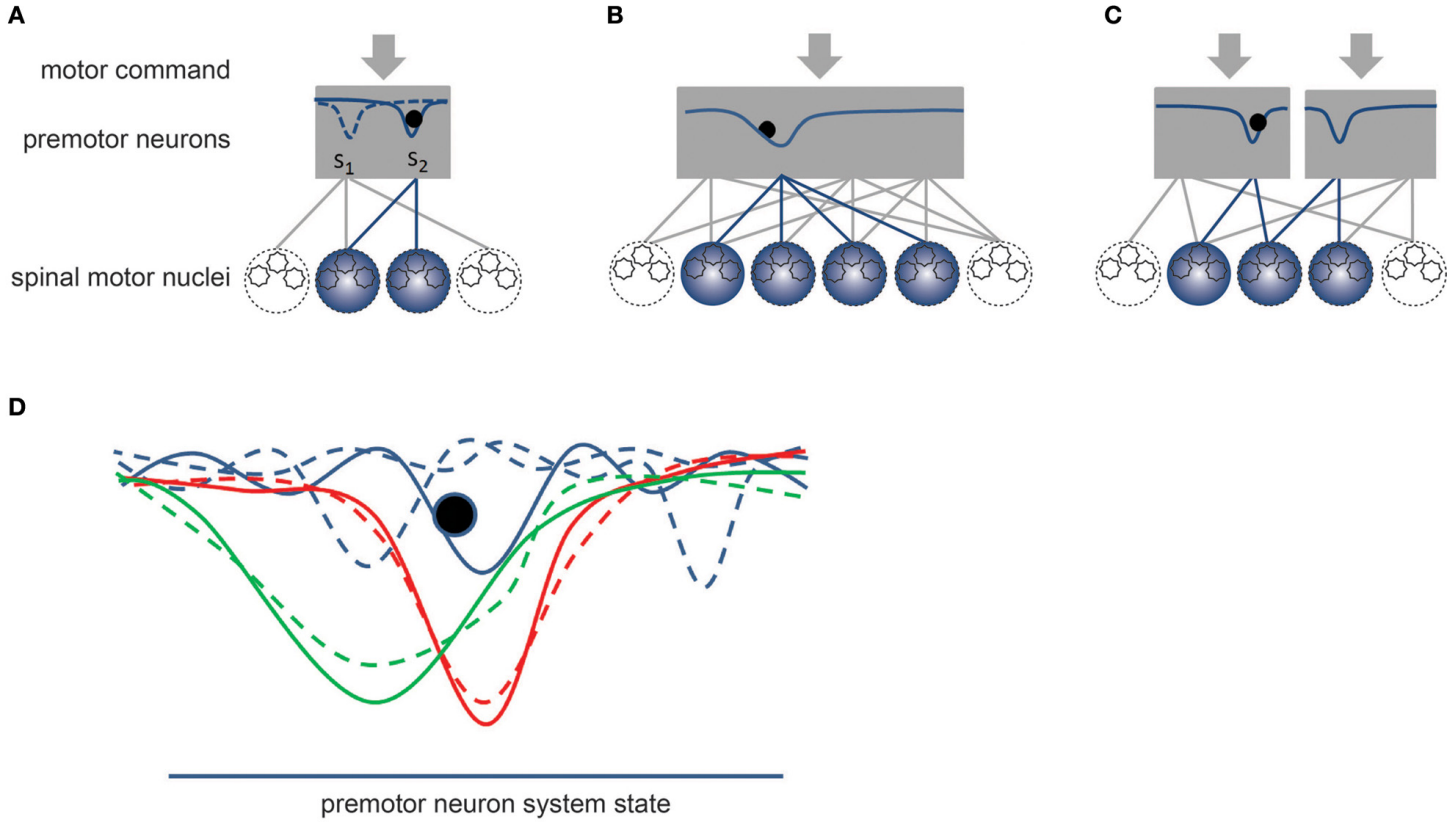
**Schematic representation of synergies.** The gray rectangles represent the dynamic system formed by pools of premotor neurons. By definition, a synergy would correspond to a stable state of the dynamical system. **(A)** The solid curve within the rectangle represents the potential field of the system, which is controlled by the descending motor commands. The dashed curve corresponds to another potential field that might be established by a different set of motor commands. Once a synergy is enabled, the current state (black sphere) of the system converges toward the stable state of the system, which establishes a specific coordination pattern or synergy between the alpha-MNs belonging to different motor nuclei. **(B)** Example of a synergy involving a larger number of spinal motor nuclei. **(C)** Example of compartmentalization of premotor neuronal pool allowing for the simultaneous activation of several synergies. The divergent connections from premotor to alpha-MNs combine together the contributions of these pools. **(D)** Effect of learning. Blue curves represent a set of unstable strategies. A slight variation in the motor commands could easily change the dynamic of the system (see dashed curves). The red and green curves represent two stable synergies, in which the impact on the system dynamics of small variations of the motor commands is smaller (dashed curves). A narrow valley (red curve) would correspond to a fixed synergy while a wider valley (green curve) would correspond to a more flexible synergy.

Overall, the pool of premotor neurons seems to include all the necessary circuitry to function as a dynamical system endowed with one or, possibly, several stable states corresponding to specific patterns of muscle activation or synergies. Figure 4 provides a schematic representation of this theoretical framework, where the descending motor commands would control the dynamics of the system by controlling the shape of its potential function. We do not argue that the dynamics of the pool of premotor neurons can always be adequately represented by a potential function but this example is used here because it is sufficient to describe the concept of synergies as we conceive it. According to this view, a synergy corresponds to a pattern of muscle activation (Figure 4A). The number of premotor neurons and motor nuclei involved in a synergy might vary (Figure 4B). For example, the coordination of contact forces between five digits might require that a larger number of motor nuclei are coordinated together. This view is also compatible with the concept of motor primitives, which could correspond to a set of synergies that are simultaneously activated to control the hand. In this case, separate sub-pools of premotor neurons would form distinct dynamical systems that could each determine a specific muscle activation pattern or motor primitive (Figure 4C). The convergent input of these pools of excitatory and inhibitory premotor neurons would summate in the alpha-MNs of the different muscles, hence determining the pattern of muscle activation (see also Figure 3). This scheme might allow the CNS to control hand muscle activation by combining together various activation patterns (see the above-discussed force mode hypothesis and kinematic synergies).

According to this view, the degree to which a specific synergy (under a given task condition) is stable (or the degree to which it will appear fixed) may reflect the degree to which this synergy is learned in the circuitry. In absence of well-defined synergies, the dynamic of the system formed by the pool of premotor neurons would correspond to a relatively flat potential function with multiple local minima. Sensory feedback or noise could disruptively affect the state of premotor neurons and spinal motor nuclei, which would have to be precisely controlled from higher centers. Another possibility in novel situations with no pre-learned movement patterns is that subjects may resort to using and combining pre-existing synergies. In this case, poorly learned tasks may therefore be associated with a larger degree of variability in the synergy selection, but this would not necessarily imply that the processing at the premotor neuron level itself is in an unstable state.

In contrast, stable synergies would correspond to deeper valleys of the potential field (see Figure 4D). By definition, these deeper and possibly wider valleys make it less likely that the system will exit from this stable state. Interestingly, it might be possible to relate the degree of flexibility of a synergy to the width of the valley: a narrow valley would correspond to fixed synergy while a wider valley might correspond to a more flexible synergy. A narrow valley would result in a more rigid coordination pattern, less susceptible to disruptions or noise, thereby appearing fixed. In contrast, the state of the system and, therefore, the coordination pattern of a flexible synergy might fluctuate as a result of sensory feedback. In particular, a wide valley synergy might accommodate in a flexible manner perturbations or unexpected events such as grasping a bigger than expected object or an object slip during manipulation.

If the spinal interneuron pool is involved in the synergy formation mechanism, the types and patterns of sensory feedback may strongly influence the synergy. Spinal premotor neurons in many cases receive monosynaptic feedback from peripheral sensors and hence provide a very fast pathway for sensory feedback to affect the selection of activated alpha-MNs with short temporal delays. The strong drive provided by sensory feedback cannot be ignored by the higher centers that control the dynamic of the spinal circuitry underlying the formation of synergies. It is conceivable that the higher centers might set up the dynamic of this system in such a manner as to switch the motor output or the coordination pattern when some external event happens. For example, such mechanisms could be used to control the various phases of a lift in an automated manner (Johansson and Flanagan, 2009).

According to the ideas presented in this discussion, the direct corticomotoneuronal connections provided by some of the layer V neurons (i.e., the monosynaptic connections from the motor cortex to the alpha-MNs) would have almost no part in the formation of hand synergies because these connections do not influence directly the circuitry formed by the spinal premotor neurons. They also lack the inhibitory component which is necessary to prevent their widely divergent excitatory connections from resulting in the inadvertent activation of all hand muscles. This view, however, does not negate that these well-documented connections play an important role in the control of the hand. This role might include, for example, a direct control of the hand movement when hand synergies are absent or not activated. These connections could also allow the cortex to control or modulate the effect of hand synergies by applying a bias to specific finger muscles when necessary. However, due to the divergence also of the direct corticomotoneuronal connections, this role, too, may be exerted in a synergistic fashion (Shinoda et al., 1979, 1981; Fetz and Cheney, 1980; Cheney and Fetz, 1985; Cheney et al., 1985; Buys et al., 1986; Lemon and Mantel, 1989; Bennett and Lemon, 1994; McKiernan et al., 1998; Rathelot and Strick, 2006, 2009). How cortical and spinal circuits interact in selecting and shaping hand synergies remains a major question in motor neuroscience. Nevertheless, the proposed framework capitalizes on recent developments in our understanding of the spinal machinery suggesting that the spinal circuits could explain many experimental observations about synergies as revealed by studies of hand kinematics, kinetics, and EMG. Note that our framework incorporates the notion of variable time shifts of individual synergies that is central to the notion of “time-varying” synergies [for review see Bizzi et al. (2008)]. Specifically, in our proposal, shifts in the temporal relation among synergies would result from time-varying interactions between cortical inputs to pre-motor neurons and afferent signals from the periphery.

If one accepts this view of synergy control, a number of interesting consequences unfold.

First, for a synergy which is often used, the premotor circuitry could adapt in a way that allows a fairly wide range of spatiotemporal patterns in the motor commands and still produce the same synergy. Compatible with this notion is the effect that cortical microstimulation in various places in the motor cortex of the monkey, which would be expected to evoke a large variety of spatiotemporal patterns in the corticospinal tract, evokes just a handful of synergies (Overduin et al., 2012). In other words, in the framework that we propose the brain might not have to control the state of the premotor and motoneurons with the same precision as it would in absence of synergies. The synergies, as already postulated by Bernstein, would greatly simplify performance of a given task by the CNS while minimizing the effects of noise from higher centers on motor output.

Secondly, with respect to the long-standing question about the degree of flexibility of synergies, the flexibility of the synergy would be related to the shape of the potential field of the dynamical system, which suggests that a whole range of possibilities exists between a deep valley (a fixed synergy) and a flat potential which would correspond to the absence of any synergy. This scheme also allows for the possibility that synergies could be fixed along some directions and flexible along other ones (for example, see the unconstrained manifold hypothesis).

Admittedly, this view is still more of a framework than a detailed model. Nonetheless, it clarifies the respective roles that the spinal circuitry, motor commands, sensory feedback, and learning could play in the formation of synergies. This theoretical framework remains to be thoroughly tested in experiments. In particular, numerous proposed circuitry mechanisms in brain synergy control remain almost completely unexplored. Altogether, these considerations point to the need for designing complementary behavioral, neurophysiological, and modeling studies to more conclusively demonstrate the interplay among the above factors underlying the modulation of synergies.

### Conflict of interest statement

The authors declare that the research was conducted in the absence of any commercial or financial relationships that could be construed as a potential conflict of interest.

## Footnotes

^1^In-hand manipulation of objects are not reviewed here as they have not been studied to the same extent.
